# Different Factors Determining Motor Execution and Motor Imagery Performance in a Serial Reaction Time Task with Intrinsic Variability

**DOI:** 10.3390/brainsci16020147

**Published:** 2026-01-29

**Authors:** Patricia Silva de Camargo, Paulo Roberto Cabral-Passos, André Frazão Helene

**Affiliations:** 1 Institute of Psychology, University of São Paulo, São Paulo 05508-030, Brazil; 2 Department of Physiology, Institute of Biosciences, University of São Paulo, São Paulo 05508-090, Brazil; andrefrazao@gmail.com; 3 Institute of Mathematics and Statistics, University of São Paulo, São Paulo 05508-090, Brazil; paulorcpassos@gmail.com

**Keywords:** motor imagery, motor execution, serial reaction time task, probabilistic motor learning, sequence structure learning

## Abstract

**Background/Objectives:** Motor imagery (MI) is the mental practice of motor actions with temporal dynamics and neural features in common with motor execution (ME). Although MI can improve motor performance, it remains unclear how closely performance-related signatures of MI resemble those of ME during learning, particularly in tasks with intrinsic variability. This study investigated similarities and differences between MI and ME during a probabilistic sequence-learning task. **Methods:** Participants performed a finger-tapping serial reaction time task in either a motor execution (ME; *n* = 10) or motor imagery (MI; *n* = 10) condition. The task consisted of 750 auditory stimuli mapped to right-hand finger movements and generated by a probabilistic sequence with deterministic and variable events. Reaction times were analyzed using ANOVA designs to assess the effects of Group, Block, Event Type, and the Last Variable event. **Results:** The MI group showed a significant reduction in reaction times across blocks (*p* < 0.001), indicating learning-related performance improvement, whereas no block-wise improvement was observed in the ME group. Both groups were sensitive to the probabilistic structure of the sequence, with reaction times differing across event types. A significant Group × Event interaction (*p* < 0.01) indicated distinct performance signatures for MI and ME. In both groups, reaction times were modulated by the last variable event. **Conclusions:** Motor imagery supported learning in a probabilistic sequence task but was influenced by factors distinct from those governing motor execution, suggesting partially different underlying mechanisms.

## 1. Introduction

Motor imagery corresponds to the mental practice of a motor task, involving both visual and kinesthetic components [1,2]. Evidence shows that motor imagery engages several brain regions whose activation patterns resemble those observed during actual motor execution [3,4,5,6]. Besides that, the duration of motor imagery and motor execution is also correlated [7]. Therefore, it is reasonable to assume that motor imagery and execution share similar mechanisms and might lead to similar outcomes, such as learning [1,2]. This assumption is supported by improvements in performance shown in motor imagery studies [8,9,10,11].

One theoretical framework aiming to explain how these shared characteristics may give rise to learning effects in motor imagery is the motor emulation theory (MET). From the perspective of this framework, the essence of mental practice is to induce an experience without the original input of external stimuli [12]. Motor *emulation* theory (MET) proposes that the brain presents emulators and plan encoders. The purpose of the former would be to provide predictions to the latter, enabling corrections and adjustments [13]. According to the MET, these emulators still provide forward models of sensory predictions during motor imagery [13,14,15]. In both motor execution and motor imagery, the predictions from the emulators are based on somatosensory consequences estimated from the efferent copy of motor commands [16]. Efferent copies contain information about the motor execution, sensory consequences, and location of the action (afferent proprioceptive input), and they are directly involved in error processing, motor control, motor execution, visual and auditory perception, posture, and tactile perception [17]. While prior studies have established functional overlaps between motor execution and imagery [18,19], our work uniquely examines how these processes adapt to intrinsically variable sequences, revealing distinct temporal signatures that inform both theoretical models and applied technologies (e.g., BCIs).

To tackle learning associated with motor imagery, in the present article, we use a paradigm introduced by Duarte et al., 2019 [20] to study how humans learn regularities in sequences of events with unpredictable steps. Humans must often deal with sequences that are unpredictable at some level and have to make decisions based on them, therefore this paradigm has a strong ecological appeal. In this paradigm, stimuli are generated based on a probabilistic context tree model, henceforth referred to just as a context tree. Basically, a context tree is a set of contexts and associated probability measures. In a sequence following a context tree, each new element is generated based on a suffix of the past sequence, which may vary in length [21]. In turn, each context of the sequence presents an associated set of probability measures. If the sequence is composed of an alphabet, say A, B, and C, these probabilities dictate how often A, B, and C come after the context. A sequence can be infinitely long but with a finite number of contexts of finite length and for each context, the probabilities are fixed. So learning the sequence translates to inferring the contexts and probability measures of the sequence [22]. In Duarte et al. (2019) [20], individuals were exposed to sequences of auditory events generated by a context tree. The structure of the sequence was decoded from their brain activity, demonstrating that the brain learns the sequence structure; that is, brain activity related to a given stimulus varies as a function of its preceding context, reflecting the regularities described in the context tree. Building on this, we hypothesize that in serial reaction time tasks, a similar signature associated with the stimuli will be reflected in reaction times, both during motor execution and motor imagery.

Motor imagery may function similarly to simulation, which involves practicing a scenario with fewer variables. Our purpose was to investigate how closely the performance signatures of real execution and motor imagery relate to each other during a learning task, given the reduced complexity of motor imagery. The learning task was a serial reaction time task, in which participants performed finger tapping according to a sequence of stimuli generated by a context tree. Two groups were included: motor execution and motor imagery. We hypothesized that (1) participants in the motor imagery group would improve performance during training and (2) the duration of motor imagery would be influenced by factors distinct from those determining reaction times in motor execution.

## 2. Materials and Methods

### 2.1. Participants

The study included two groups of healthy right-handed participants capable of performing imaginative tasks: the motor execution group with 10 participants (6 women, mean age = 27.1 ± 4.18 years) and the motor imagery group with 10 participants (5 women, mean age = 26.4 ± 4.93 years). Refer to Table 1 for detailed characteristic information about all the volunteers. The experiment occurred at the Cognition Science Laboratory in the Institute of Biosciences (IB-USP) of the University of São Paulo. All participants were either undergraduate or graduate students at the University of São Paulo. The study was conducted in accordance with the Declaration of Helsinki and approved by the local ethics committee (CAAE 15274718.8.0000.5464, Institute of Biosciences at the University of São Paulo—IB-USP), and all participants provided informed consent.

Participants were selected according to the following criteria. They should be over 18 years old, right-handed, and be capable of performing imaginative tasks. They should also present no history of neurological dysfunctions; spinal fractures; or orthopedic, vascular, or muscular dysfunctions affecting the upper limb. The handedness dominance was evaluated using the *Edinburgh Handedness Inventory* validated for Brazilian Portuguese [23,24]. Participants with scores greater than 50 were considered right-handed. The ability to perform imaginative tasks was evaluated using the *Kinesthetic and Visual Motor Imagery Questionnaire—KVIQP-10, Brazilian version* [25]. Each scale in the questionnaire (both dominant and non-dominant sides of the body) has a score range from 9 to 45, with higher scores indicating a greater ability in motor imagery. Participants with scores greater than 25 were included in the sample.

### 2.2. Experimental Task

The experiment used *Psychtoolbox-3 (Psychophysics Toolbox Version 3—PTB-3, CA, USA)* software version 3.0.17 Beta to present auditory stimuli. The auditory stimuli set were labeled as one, two, and three indicating which action the participant should take. The auditory stimuli consisted of recordings from a single Brazilian Portuguese speaker, captured using digital audio recording software (Audacity, version 3.0.0) with a USB conventional microphone (Blue Yeti, Logitech, Palo Alto, CA, USA), and were presented using Samsung in-ear headphones (Samsung model EO-EG920B, China). The volume was adjusted individually for each participant to ensure comfort.

The experiment was a serial reaction time task. The sequence of stimuli consisted of three possible stimuli: the words for the numbers 1, 2 and 3 in Brazilian Portuguese; that is, *um*, *dois* and *três*, respectively. Upon hearing a stimulus, participants were instructed to press the corresponding key on a standard keyboard using a specific finger: 1 (index finger), 2 (middle finger), and 3 (ring finger). The keys were mapped as follows: left arrow for 1, down arrow for 2, and right arrow for 3.

**Figure 1:**
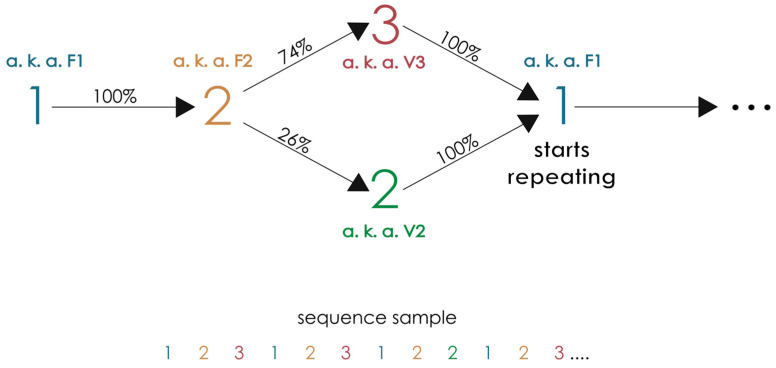
Graphical representation of the sequence of stimuli in the task. The sequence of auditory, where “1” and “2” are fixed events with a 100% probability of occurrence (referred to as F1, blue, and F2, orange, respectively); the occurrence of “2” (26% probability) or “3” (74% probability) are variable events (V2, green, and V3, red, respectively). The samples of the sequence are indicated with the corresponding colors described above.

The transitions between stimuli were determined by a context tree, a universal system for compressing sequence information that allows both deterministic and probabilistic transitions [23]. The context tree used in this study was previously tested to verify learnability in [26], and its transition probabilities are illustrated in Figure 1. Context trees similar to this one were used in other studies [27,28], which demonstrated that behavioral responses, such as reaction times, are modulated by regularities embedded in the stimuli, even in the presence of intrinsic variability. This modulation extends beyond simple stimulus–response associations, indicating that participants learn the underlying sequence structure, which becomes imprinted in their behavioral responses. Additionally, the intrinsic variability of the stimuli emulates the unpredictability observed in natural environments, making the paradigm particularly suitable for investigating real-world sequence learning.

Specifically, in the context tree of the present study, from stimulus 1, the transition is deterministic: the next stimulus is always 2 (100% chance). From stimulus 2, the transition depends on the preceding stimulus. If 2 is preceded by 1, the next stimulus can be 3 (74% chance) or another 2 (26% chance). If 2 is preceded by another 2, the transition is deterministic: the next stimulus is always 1 (100% chance). To distinguish between deterministic and non-deterministic conditions, we used a coding system. F2 indicates that the stimulus 2 is deterministic (F for fixed). V2 indicates that the stimulus 2 is non-deterministic (V for variable). For example, a possible sequence realization could be: 1, 2, 3, 1, 2, 2, 1, 2, 3. For a detailed explanation of how sequences are generated using context trees, we refer the reader to Cabral-Passos et al., 2024 [27].

### 2.3. Experimental Protocol

Participants performed the task seated in front of a 17-inch LCD monitor (Samsung, 60 Hz refresh rate, China) that was positioned 60 cm away and aligned to the participant’s eyes. The room in which the task was performed remained silent throughout the task. The researcher provided the instructions and clarified any questions to the participants before the task initiation.

#### 2.3.1. Familiarization Phase

The phase described was designed solely for familiarization with the task and was conducted in the same way for both groups of participants. The participants were presented with an auditory stimulus of a number (1, 2, or 3) during each trial. Each number corresponds to a specific finger of the right hand, with the index finger associated with the number 1, the middle finger with the number 2, and the ring finger with the number 3. Participants were instructed to press the corresponding key on the keyboard as quickly as possible upon identifying the number. Participants were informed that if they pressed the key before the auditory stimulus, a beep would sound indicating that the current trial was invalid before moving to the next trial.

**Figure 2:**
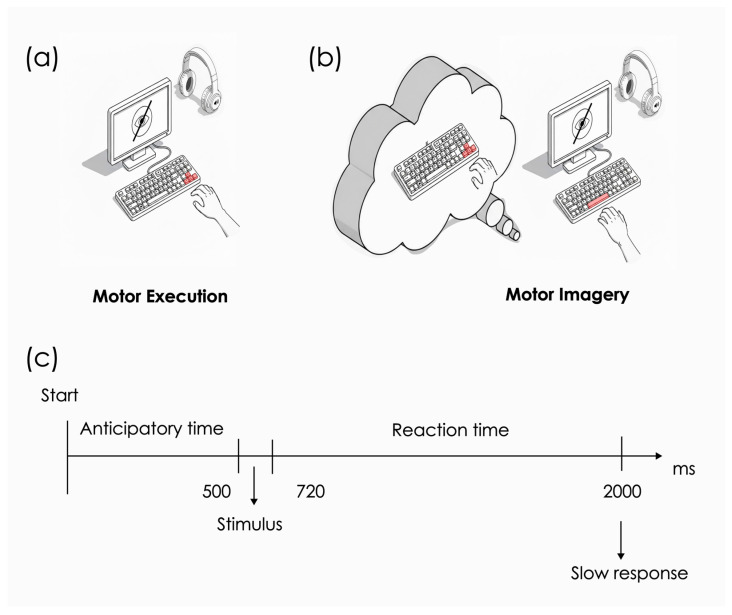
Schematic representation of each trial under the conditions of motor execution and motor imagery. Schematic representation of each trial under the conditions of (**a**) motor execution and (**b**) motor imagery. (**c**) Trial schematic arrangement containing the anticipatory time (500 ms before the stimulus), the average duration of the auditory stimulus (220 ms), the reaction time that depends on the individual’s motor response, and the duration of what was considered a slow response (2000 ms). The keyboard keys used to provide the response in each condition have been highlighted in red.

The reaction time for each trial was defined as the time from the end of the auditory cue until the beginning of the motor response (see Figure 2). This phase consisted of only six trials, with two trials associated with each of the fingers and a randomly assigned order.

#### 2.3.2. Test Phase

During the test phase, participants were presented with 750 auditory stimuli divided into 5 blocks of 150 trials each. Table 2 shows the frequency associated with F1, F2, V2, and V3 for each block. Each block lasted on average approximately 3 min across participants, varying with each participant’s individual reaction times. Between blocks, participants were allowed to take a rest period of their choice to minimize fatigue and maintain attention. Reaction time for each trial was defined as the interval from the end of the auditory stimulus until the initiation of the participant’s motor response (or the imagined response in the motor imagery group). This block structure and session duration were chosen to provide a sufficient number of trials for statistical analysis while minimizing fatigue, in line with previous studies using serial reaction time tasks.

Participants were instructed to keep their eyes closed until the end of each block indicated by the sound of two consecutive “beeps”. In the motor execution group, participants were required to press the correct key with the corresponding finger of the right hand immediately after identifying the number (1, 2, or 3). There was no register of any key presses during the stimulus presentation. If a participant missed the key press or pressed the wrong key, the program waited for the correct response before proceeding to the next trial (mean and standard deviation of missed key presses: 0.001 ± 0.033). In the motor imagery group, participants were instructed to imagine the visual and kinesthetic sensory inputs of the finger-tapping execution from a first-person perspective. This perspective refers to imagining the action from one’s own point of view, as if actually performing the movement, emphasizing internal visual and kinesthetic sensations, whereas the third-person perspective involves imagining the action from an external observer’s viewpoint. The first-person perspective was adopted because of evidence that imagination from this perspective appears to be limited by constraints similar to those of execution [29,30]. Once the motor imagery was completed, they were instructed to press the “spacebar key” with the left index finger as illustrated in Figure 2.

### 2.4. Statistical Analysis

All statistical analyses were performed using the Python (*version* 3.13) programming language. Additionally, Statsmodels and Pingouin Python packages were used for statistical tests [31,32]. Two-way ANOVA designs were used to test differences in the distributions of mean reaction times across blocks, events, and groups. The first design examined the within-group effects of Block and Event for each group separately. The second design assessed the between-group effects of Group and Event, collapsing the Block factor to improve statistical power and interpretability. Follow-up pairwise comparisons were conducted to identify specific differences between conditions. The mean reaction times during the test phase were compared across groups in a 2-way mixed ANOVA. “Group” was considered as a between-subjects factor, while “Block” and “Event” were regarded as within-subjects factors. Only correct reaction times smaller than 2 s were included in the analysis. All code, output and description is available in the following link: https://github.com/PauloCabral-hub/third_party_work/blob/main/data_analysis_routine.ipynb (accessed on 27 January 2026). For further details on the statistical analysis including power analysis, see the Supplementary Materials [33,34,35].

## 3. Results

The results are presented in three parts for each analysis: (1) a description of the input to the analysis design, (2) the statistical results, and (3) a concise, objective summary of the conclusions directly derived from the data.

### 3.1. Performance Analyses Associated with the Probabilities of the Events

**Figure 3:**
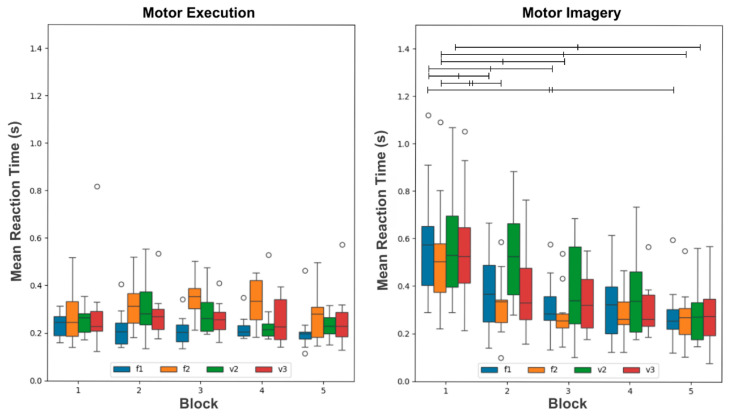
Performance analysis (mean reaction times) of motor execution and motor imagery as a function of Block and Event. Distributions of the mean reaction times for the events F1 (blue), F2 (orange), V2 (green), and V3 (red) across blocks for the motor execution and motor imagery groups. The line between the first and third quartiles indicates the median value. Bars on top of the distributions indicate the statistical significance of the follow-up pairwise comparison tests (thin bars for *p* < 0.05 and thick bars for *p* < 0.01). Outliers are indicated as small circles.

Mean reaction times were used as indirect measures of performance in all analyses below. Figure 3 shows the distribution of the mean reaction times for F1, F2, V2, and V3 events in each of the five blocks of the motor execution (left) and motor imagery (right) groups. For both groups, a two-way repeated-measures ANOVA with rank-transformed values was used to verify significant differences across blocks and events. The procedures for verifying test assumptions and corrections in cases of violation can be found in the Supplementary Materials.

For the motor execution group, the two-way repeated-measures ANOVA revealed significant effects for the factor Event (F_3, 27_ = 5.569, *p* < 0.012), but not for Block (F_4, 36_ = 1.252, *p* = 0.310) and no interaction between Block and Event (F_12, 108_ = 1.095, *p* = 0.372). Follow-up pairwise comparison tests showed differences for the Events F1 and F2 (*p* < 0.01) (see Supplementary Table S1 for details).

For the motor imagery group, the ANOVA revealed significant effects of Block (F_4, 36_ = 17.456, *p* < 0.001) and Event (F_3, 27_ = 6.095, *p* < 0.016), but no interaction between Block and Event (F_12, 108_ = 1.734, *p* = 0.189). Follow-up pairwise comparisons with the respective Bonferroni corrected *p*-values are available in the Supplementary Table S2 [36]. Significant pairwise comparisons for specific distributions are depicted in Figure 3 as bars of different thicknesses, thin bars for *p* < 0.05 and thick bars for *p* < 0.01. The mean reaction times for V2 and V3 decreased across the blocks. The mean reaction times in V2 were higher during blocks, potentially due to the smaller probability of this event.

Reaction times for execution tend to become shorter as the experiment progresses in paradigms similar to the one employed here. However, no block effect was seen in our execution group. To further investigate if this effect was somehow masked by some aspect of our design, the following analysis was performed. For each group and block, mean reaction times were calculated for sub-blocks of 50 trials, without superposition and disregarding the stimulus type. Therefore, three sub-blocks were considered, totaling the 150 trials of each block. A linear regression was imposed on the mean reaction times of the sub-blocks of each subject so as to retrieve the regression’s slope. If shorter reaction times were more common closer to the end of each block, the distribution of the slopes should be negative. To verify that hypothesis, a one sample, two-sided *t*-test was employed to the distribution of the slopes of each block and group. For both groups, the slopes of the first three blocks were more negative (see Supplementary Figure S1). For the motor imagery group, the slopes of the last two blocks were also more negative, however for the motor execution group, the slopes were more closely distributed around zero. The test indicated that the slopes of the motor execution group were significantly negative only in the second block (*t* = −2.365, df = 9, *p* = 0.04). For the motor imagery group, the slopes were significantly negative only for the first block (*t* = −3.173, df = 9, *p* = 0.01). These results indicate that a reduction in reaction times is present within blocks for both groups, although not across blocks. This reduction is seen first in the motor imagery group, that is, in the first block, and then in the motor execution group in the second block.

In summary, mean reaction times decreased across blocks for the motor imagery group, indicating improved performance across the experiment. However, there was no evidence that reaction times decreased across blocks for the motor execution group, although there was evidence of a decrease within blocks. As for the sensitivity to sequence structure, mean reaction times for both groups appear to be sensitive to it.

**Figure 4:**
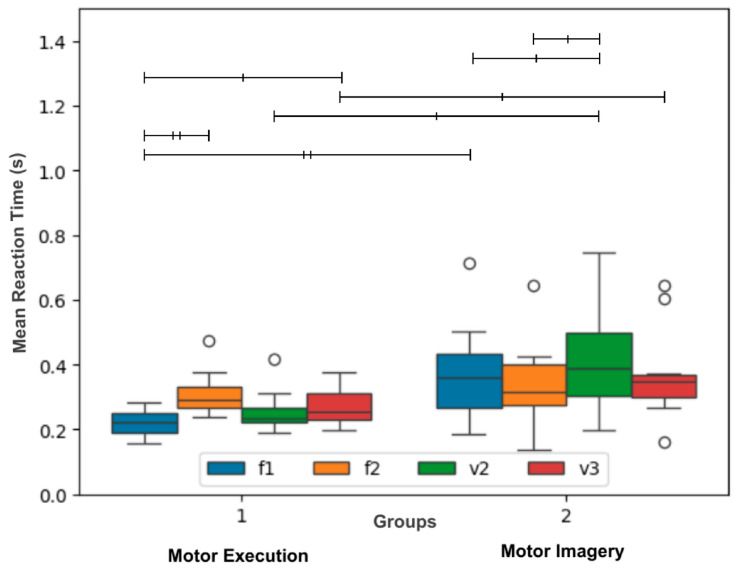
Performance of the motor execution and motor imagery groups as a function of Event. Distribution of the mean reaction times for events F1 (blue), F2 (orange), V2 (green), and V3 (red) across groups (motor execution—left, motor imagery—right). The line between the first and third quartiles indicates the median value. Bars on top of the distributions indicate the statistical significance of the follow-up pairwise comparison tests (thin bars for *p* < 0.05 and thick bars for *p* < 0.01). Outliers are indicated as small circles.

A two-way mixed ANOVA was used to compare both Groups across Events. Figure 4 shows the distributions of mean reaction times of motor execution (left) and motor imagery (right) disregarding block information. The two-way mixed ANOVA indicated significant differences for the between-subjects factor Group (F_1, 18_ = 7.353, *p* < 0.05), but not for the within-subjects factor Event (F_3, 54_ = 3.849, *p* > 0.05). The test also indicated significant interaction between Group and Event (F_3, 54_ = 7.172, *p* < 0.01). Follow-up pairwise comparisons with the respective Bonferroni corrected *p*-values are available in the Supplementary Table S3. Significant pairwise comparisons for specific distributions are depicted in Figure 4 as bars of different thicknesses, thin bars for *p* < 0.05 and thick bars for *p* < 0.01. It is evident from the figure that except for F2, all events differed between groups. On the other hand, only some events differ within each group.

In summary, the evidence shows that mean reaction times differ from one group to another. This was expected given the previous results. Furthermore, the significant interaction shows that the mean reaction times of each group exhibit different signatures associated with each stimulus type.

### 3.2. Performance Analysis as a Function of the Impact of Last Variable Event

**Figure 5:**
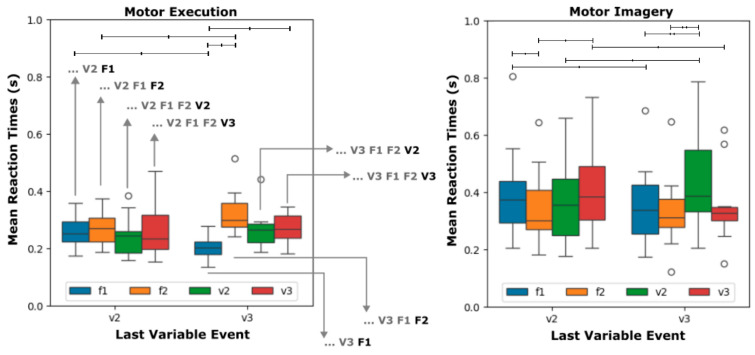
Performance of the motor execution and motor imagery groups for each Event as a function of Last Variable Event. Distributions of mean reaction times for the events F1 (blue), F2 (orange), V2 (green), and V3 (red) after the last variable event (V2 and V3) for the motor execution and motor imagery groups. An arrow was drawn from each distribution presented in the motor execution group to illustrate the cases considered. The three dots indicate the distant past of the sequence, and the gray stimuli indicate the recent past starting at the Last Variable Event and ending at the stimulus corresponding to the distribution (black). The line between the first and third quartiles indicate the median value. Bars on top of the distributions indicate the statistical significance of the follow-up pairwise comparison tests (thin bars for *p* < 0.05 and thick bars for *p* < 0.01). Outliers are indicated as small circles.

Figure 5 shows the distribution of mean reaction times for each Event given the immediate Last Variable Event for both groups, motor execution (left) and motor imagery (right). That is, for both groups, the four distributions on the left indicate the mean reaction times following a V2 for F1, F2, V2 (itself) and V3. On the other hand, the four distributions on the right indicate the mean reaction times following a V3 for F1, F2, V2, and V3 (itself). To test for differences in the Events across the Last Variable Event, a two-way repeated-measures ANOVA was applied to the distributions of the mean reaction times of each group. For the motor execution group, no difference was observed for the factor Event (F_3, 27_ = 3.313, *p* > 0.05) and Last Variable Event factor (F_1, 9_ = 1.009, *p* > 0.05). On the other hand, a significant interaction between both factors were observed (F_3, 27_ = 6.169, *p* < 0.01). Follow-up pairwise comparisons with the respective Bonferroni corrected *p*-values are available in the Supplementary Table S4. Significant pairwise comparisons for specific distributions are depicted in Figure 5 as bars of different thicknesses, thin bars for *p* < 0.05 and thick bars for *p* < 0.01. As depicted in Figure 5 (left), the mean reaction times for F1 are smaller after the last variable event V2 than after V3. On the other hand, the converse occurs for F2. This contrast suggests that the anticipation of the variable event in F2 may increase the reaction times.

Similarly, for the motor imagery group, no difference was observed for the factor Event (F_3, 27_ = 3.334, *p* > 0.05) and Last Variable Event factor (F_1, 9_ = 1.526, *p* > 0.05). However, a significant interaction between both factors was observed (F_3, 27_ = 13.216, *p* < 0.01). Significant follow-up pairwise comparisons with the respective Bonferroni corrected *p*-values are available in the Supplementary Table S4. Significant pairwise comparisons for specific distributions are depicted in Figure 4 as bars of different thicknesses, thin bars for *p* < 0.05 and thick bars for *p* < 0.01. Significant follow-up pairwise comparisons with the respective Bonferroni corrected *p*-values are available in the Supplementary Table S5. Significant pairwise comparisons for specific distributions are depicted in Figure 5 as bars of different thicknesses, thin bars for *p* < 0.05 and thick bars for *p* < 0.01. As depicted in Figure 5 (right), the mean reaction times for V2 are smaller after the last variable event V2 than after V3. On the other hand, the converse occurs for V3; that is, mean reaction times are larger after V2. This contrast does not appear in the motor execution group. Besides that, no differences are verified between F1 and F2 following V2 and V3.

So, in essence, the evidence on the significant interaction between Event and Last Variable Event shows that the signature associated with a stimulus type can change as a function of the last variable event in both motor execution and motor imagery.

## 4. Discussion

The current study was developed to investigate functional equivalences/differences between motor imagery and motor execution. The study provides evidence of how sequence structures with intrinsic variability are processed by the human subjects during motor imagery. Given that motor imagery enhances learning of motor sequences and benefits the development of fine motor skills [37], we aimed to investigate how motor execution and motor imagery differs during a structural learning task. We used reaction time as an indirect measure of performance. The finger-tapping serial reaction time task used followed a structure determined by the same context tree across all experimental blocks and presented events with different probabilities resulting in a sequence with intrinsic variability. The results showed that (1) performance improves in the motor imagery group in the given task and (2) the duration of motor imagery indirectly measured by reaction times is influenced by distinct factors to those of motor execution.

The current study showed that the mean reaction times of the motor imagery group decreased across the task blocks (Figure 3, right). Surprisingly, there was no significant reduction in reaction times across blocks for the motor execution group (Figure 4, left), just within blocks. Several factors may contribute to the difference in results between the two groups: (1) the absence of somatosensory feedback during the three-finger-mapped movements in the motor imagery (MI) group; (2) differential effects of fatigue on execution versus imagery; (3) differences in the time required to reach a performance plateau between execution and imagery; (4) differences in susceptibility to attentional fluctuations; and (5) the simplicity of the task. Some of these factors are inherently difficult to compare between the two practices; for instance, factor (1) is absent in execution, whereas factor (5) may be equal at a first glance. Regarding (3), the time to plateau is likely longer in imagery. As noted by Driskell, Copper, and Moran, 1994 [8], significant effects of mental practice on execution performance typically appear only after approximately 20 min of imagery. As evidence suggests that ME and MI have roughly mirrored times [38], it is reasonable to expect that the effect of MI on itself, as we evaluate in the present study, does take long to appear. Although the minimum time for improvement in motor execution only is not consistently reported, some calculations may help us estimate it. For example, Nissen and Bullemer, 1987 [39] show measurable improvement as early as the second block of trials. Thus, assuming an overestimated reaction time of 500 ms across 100 trials per block and accounting for the inter-stimulus intervals, performance improvements did occur in less than 2 min, which is less than the time of one block in the present study. While this may be considered a very rough estimate, it does suggest that improvements and time to plateau often take longer in imagery than in execution.

With respect to factor (2), our results demonstrate a consistent reduction in reaction times for the motor imagery (MI) group, with no evidence of fatigue-related performance decrement over the course of the task. This pattern contrasts with previous findings, which reported significant increases in motor imagery duration due to fatigue following repeated imagery trials with interleaving of actual execution [40]. One plausible explanation for this discrepancy may relate to task-specific demands: while Rozand et al., 2016 [40] employed whole-arm pointing movements, our study utilized a fine motor, finger-based sequence task. Notably, task duration alone is unlikely to account for the observed differences, as participants in the present study performed 150 imagined movements per session, exceeding the 100 trials in the [40].

With respect to attentional fluctuations (factor 4), further investigation using dedicated measures of brain activity will be necessary. Both fatigue- and attention-related effects are therefore likely to require more sensitive experimental paradigms and measurement techniques to be reliably quantified. The stimulus sequence used in this study was selected for its ease of learning by most volunteers, facilitating comparison across studies. A similar sequence was used by Cabral-Passos et al., 2024 [27], where participants demonstrated a high rate of correct predictions (see paper Figure 4), supporting the suitability of this approach. This strategy was chosen in light of the ongoing lack of consensus on how to measure sequence complexity without relying on participants’ explicit reports [28]. Nonetheless, since sequence learning can occur without conscious awareness see [41] for a review), it remains crucial to develop objective metrics for assessing sequence complexity.

In our protocol, participants were instructed to initiate motor imagery by the sound that indicates which action should be imagined. In turn, to indicate the end of the motor imagery they were asked to actually perform a motor action. We acknowledge that using a motor action to indicate the end of the motor imagery may introduce some time variability to the responses, given it can be considered as switching tasks in a small time interval [42]. However, we should highlight that in an important study conducted by Decety, Jeannerod, and Prablanc, 1989 [43], subjects timed their motor imagery by activating a stopwatch to indicate the beginning and end of the mental simulation, which is very similar to the procedure here, and were able to show a strong correlation between the time of the execution and motor imagery of the same task. In fact, the use of a motor action to time motor imagery is also used in more recent studies, despite the evidence suggesting that motor actions following motor imagery are differently modulated compared to when following another motor action [44,45]. These studies report that whatever effector is used to time the motor imagery, it is subject to the inhibition of the whole motor system (termed global inhibition), but when the same effector is involved in both motor execution and motor imagery an effector-specific inhibition also takes place [45]. That way, since global inhibition seems inevitable, the choice of the left hand to time the motor imagery in our protocol may be viewed as an appropriate choice. However, since then, other methods have been employed for timing it with possible better results [46,47].

The two-way repeated-measures ANOVA applied to the data in Figure 3 indicated that both groups were affected by the probabilistic structure of the sequence. This is even more evident from the results in Figure 4. Also, despite the correlation between the duration of motor execution and motor imagery presented in Papaxanthis et al., 2002 [7], the two-way mixed ANOVA applied to the data in Figure 4 indicated significant interaction between the factors Group and Event. This indicates that the mean reaction times have a different behavior in each group. Interestingly, the mean reaction times of F1 and F2 differ in the motor execution group, but not in the motor imagery group. One possible explanation for this finding is that the expectation regarding the next event, V2 (26%) or V3 (74%), is more pronounced in the motor execution group given the associated, more-stimulus-specific motor preparation. This aligns with results that illustrate that although motor execution and motor imagery share mechanisms across their course of development [48,49], their differences are relevant and should be further investigated. For example, in Ruffino, 2021 [50] both motor execution and motor imagery led to performance gains in a motor skill, but the effects deteriorate less across the course of 6 h for the motor imagery group. Besides that, in Carrillo-de-la-Peña et al., 2008 [51], although the same activation of cortical motor-related areas are seen in both motor execution and motor imagery, the characteristics of their signature shows substantial differences.

Another important finding of the current study is related to the data in Figure 5. It showed a significant interaction between the factors Event and Last Variable Event in both groups. This result indicates that the behavior of the events also changes as a function of the last variable event in serial reaction tasks. In the motor imagery group, it is possible to see that the mean reaction times are smaller for V2 following V2 as compared to V2 following V3. Similarly, for the same group, mean reaction times are smaller for V3 following V3 as compared to V3 following V2. This suggests that motor planning is continuously taking into account the last variable event even in motor imagery.

Evidence shows that motor imagery and motor execution share brain mechanisms and provide activation of the same motor pathways [18,52,53,54]. Besides that, even the autonomic motor system [55] and spinal reflexes [19] appear to be sensitive to the practice. Despite the lack of knowledge about the details of how these systems are recruited and their inner workings during motor imagery, improvements in motor function have been found in healthy subjects [2,56,57,58,59,60] and subjects in motor rehabilitation [61] as a consequence of the practice. Therefore, it makes sense to understand how learning occurs in motor imagery as a way to develop and test motor control and motor emulation theories [13,14,62,63] that can help produce guidelines for participants to obtain the most from the practice. Our results have implications for studies that use motor imagery as a tool for specific purposes. For example, since motor execution and motor imagery seems to be influenced by different factors, training Brain–Machine Interfaces (BCIs) controlled by motor imagery must account not only for classifiers dealing with a smaller signal-to-noise ratio as compared to execution, but actually for the fact that while motor imagery and execution share some underlying dependencies, they involve signals of a fundamentally different nature. This conclusion also extends to the motor imagery used in sports [64,65,66] and clinical conditions [67,68].

In summary, our findings demonstrate that both the motor execution and motor imagery groups were affected by the intrinsically variable structure of the sequence of stimuli. The motor imagery group showed a marked improvement across blocks for the different events. Moreover, the mean reaction times showed different behavior in both groups in respect to event, block and last variable event. A limitation of the present study is the relatively small sample size, which restricts the generalizability and direct clinical interpretation of the results. Therefore, the findings should be interpreted as indicative and exploratory rather than definitive. Given the relatively small sample size of the present study, we highlight the need for additional studies with larger samples to attest to the generalizability of our results. Further studies are necessary to explore which parameters determine these differences and the specific neural activity patterns and neural substrates that differentiate motor execution and motor imagery. An interesting development of this study would be adding a follow-up evaluation to the motor imagery to assess their reaction times in the way it was adopted for the motor execution group so as to verify how performance changes as a function of the motor imagery training. Besides that, the use of cues to guide motor imagery in future studies may lead to greater accuracy and statistical power to detect differences in reaction times across conditions [69].

## Figures and Tables

**Table 1:** Demographic and questionnaire information of the participants separated into groups. Means and standard deviations (SD) are given.

	Motor Execution (Mean ± SD)	Motor Imagery (Mean ± SD)	Total (Mean ± SD)
Age (Years)	27.1 ± 4.18	26.4 ± 4.93	26.75 ± 4.46
Gender	F (6); M (4)	F (5); M (5)	F (11); M (9)
Edinburgh Inventory	82.73 ± 13.75	86.51 ± 15.3	84.62 ± 14.29
KVIQP-10V	35.1 ± 4.79	26.1 ± 8.91	30.6 ± 8.35
KVIQP-10K	32.9 ± 6.45	25.8 ± 6.98	29.35 ± 7.49

F = female; M = male; KVIQP-10V = Visual Motor Imagery Questionnaire (Brazilian version); KVIQP-10K = Kinesthetic Motor Imagery Questionnaire (Brazilian version).

**Table 2:** Quantity of each event in the sequence of auditory stimuli throughout the experimental blocks.

	B1	B2	B3	B4	B5	Total
F1	50	50	50	50	50	250
F2	50	50	50	50	50	250
V2	16	7	13	14	15	65
V3	34	43	37	36	35	185

B1–B5 = experimental blocks 1 to 5; F1 = fixed event “1”with 100% of probability of occurrence; F2 = fixed event “2” with 100% of probability of occurrence; V2 = variable event “2” with 26% of probability of occurrence; V3 = variable event “3” with 74% of probability of occurrence.

## Data Availability

The datasets analyzed for this study can be found in the: https://github.com/PauloCabral-hub/third_party_work/blob/main/Camargoetal2024_sup.pdf (accessed on 27 January 2026).

## Supplementary Materials

The following supporting information can be downloaded at https://www.mdpi.com/article/10.3390/brainsci16020147/s1: Figure S1: Regression slopes for the reaction times across sub-blocks; Table S1: Significant follow-up pairwise comparison tests for data in the left side of Figure 3; Table S2: Significant follow-up pairwise comparison tests for data in the right side of Figure 3; Table S3: Significant follow-up pairwise comparison tests for data in Figure 4; Table S4: Significant follow-up pairwise comparison tests for data in the left side of Figure 5; Table S5: Significant follow-up pairwise comparison tests for data in the right side of Figure 5; Material S1: Statistical analysis details.
