# Supplementary material for: Different Factors Determining Motor Execution and Motor Imagery Performance in a Serial Reaction Time Task with Intrinsic Variability
Source: Brain Sci. 2026 Jan 29;16(2):147. doi: 10.3390/brainsci16020147 (PMC12939135; doi:10.3390/brainsci16020147)
Supplement: Supplementary file 1 [file brainsci-16-00147-s001.zip › brainsci-4070186-supplementary.pdf]

Different factors determining Motor Execution and Motor Imagery performance in a serial reaction time task with intrinsic variability

Supplementary Figure

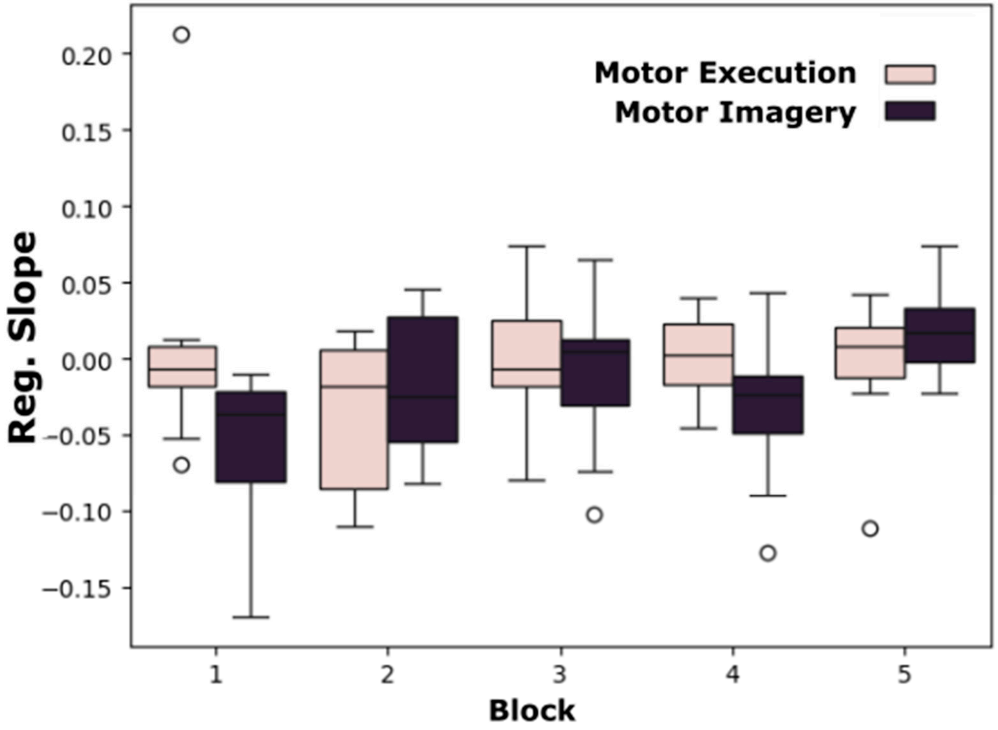

Figure S1: Regression slopes for the reaction times across sub-blocks. Distribution of the regression slopes across sub-blocks of 50 trials within each block for the Motor Execution and Motor Imagery groups.

Statistical Analysis - Tables

Table S1: Significant *follow-up* *pairwise comparison* tests for data in the left side of Figure 3.

	Contrast	Constant	A	B	*p*-corr	*hedges*
0	stim_type	-	f1	f2	0.004369	-1.536967
66	stim_type	-	f1	f2	0.004369	-1.536967

Stim_type = event type; f1 = event F1; f2 = event F2; *p*-corr = *p* value.

Table S2: Significant *follow-up* *pairwise comparison* tests for data in the right side of Figure 3

	Contrast	Constant	A	B	*p*-corr	*hedges*
3	stim_type	-	f2	v2	0.013480	-0.571729
6	block	-	1	2	0.020858	0.732498
7	block	-	1	3	0.002181	1.256330
8	block	-	1	4	0.019438	1.244827
9	block	-	1	5	0.002114	1.504281
16	stim_type * block	f1	1	2	0.026913	0.931792
17	stim_type * block	f1	1	3	0.023242	1.422443
19	stim_type * block	f1	1	5	0.005211	1.739980
26	stim_type * block	f2	1	2	0.006886	0.923347

27	stim_type * block	f2	1	3	0.016393	1.360848
29	stim_type * block	f2	1	5	0.018731	1.311399
39	stim_type * block	v2	1	5	0.029581	1.440845
56	block	-	1	2	0.020858	0.732498
57	block	-	1	3	0.002181	1.256330
58	block	-	1	4	0.019438	1.244827
59	block	-	1	5	0.002114	1.504281
69	stim_type	-	f2	v2	0.013480	-0.571729

Stim_type = event type; Block = block of the sequence; f1 = event F1; f2 = event F2; v2 = event V2; *p*-corr = *p* value.

Table S3: Significant *follow-up pairwise comparison* tests for data in Figure 4

	index	Contrast	fix	A	B	Paired	Parametric	T	dof	alternative	p-unc	p-corr	p-adjust	BF10	hedges
0	0	stim_type	-	f1	f2	True	True	-2.509989	19.0	two-sided	0.021287	0.127723	bonf	2.749	-0.486951
1	1	stim_type	-	f1	v2	True	True	-2.593998	19.0	two-sided	0.017812	0.106871	bonf	3.179	-0.335286

2	2	stim_type	-	f1	v3	True	True	-2.116157	19.0	two-sided	0.047759	0.286555	bonf	1.44	-0.330892
3	3	stim_type	-	f2	v2	True	True	0.503255	19.0	two-sided	0.620569	1.000000	bonf	0.26	0.114354
4	4	stim_type	-	f2	v3	True	True	0.745727	19.0	two-sided	0.464959	1.000000	bonf	0.298	0.158305
5	5	stim_type	-	v2	v3	True	True	0.299905	19.0	two-sided	0.767506	1.000000	bonf	0.242	0.029046
6	6	group	-	1	2	False	True	-2.711821	18.0	two-sided	0.014288	NaN	NaN	4.013	-1.161520
7	7	stim_type * group	f1	1	2	False	True	-3.712338	18.0	two-sided	0.001594	0.006377	bonf	20.775	-1.590058
8	8	stim_type * group	f2	1	2	False	True	-0.388269	18.0	two-sided	0.702371	1.000000	bonf	0.419	-0.166302
9	9	stim_type * group	v2	1	2	False	True	-3.271590	18.0	two-sided	0.004239	0.016955	bonf	9.796	-1.401278
10	10	stim_type * group	v3	1	2	False	True	-2.129001	18.0	two-sided	0.047317	0.189268	bonf	1.763	-0.911888

11	0	group	-	1	2	False	True	-2.711821	18.0	two-sided	0.014288	NaN	NaN	4.013	-1.161520
12	1	stim_type	-	f1	f2	True	True	-2.509989	19.0	two-sided	0.021287	0.127723	bonf	2.749	-0.486951
13	2	stim_type	-	f1	v2	True	True	-2.593998	19.0	two-sided	0.017812	0.106871	bonf	3.179	-0.335286
14	3	stim_type	-	f1	v3	True	True	-2.116157	19.0	two-sided	0.047759	0.286555	bonf	1.44	-0.330892
15	4	stim_type	-	f2	v2	True	True	0.503255	19.0	two-sided	0.620569	1.000000	bonf	0.26	0.114354
16	5	stim_type	-	f2	v3	True	True	0.745727	19.0	two-sided	0.464959	1.000000	bonf	0.298	0.158305
17	6	stim_type	-	v2	v3	True	True	0.299905	19.0	two-sided	0.767506	1.000000	bonf	0.242	0.029046
18	7	group * stim_type	1	f1	f2	True	True	-5.280522	9.0	two-sided	0.000507	0.006081	bonf	72.182	-1.729338
19	8	group * stim_type	1	f1	v2	True	True	-1.546007	9.0	two-sided	0.156505	1.000000	bonf	0.77	-0.612307

20	9	group * stim_type	1	f1	v3	True	True	-2.488823	9.0	two-sided	0.034489	0.413862	bonf	2.34	-0.919687
21	10	group * stim_type	1	f2	v2	True	True	1.963194	9.0	two-sided	0.081226	0.974715	bonf	1.228	0.870201
22	11	group * stim_type	1	f2	v3	True	True	1.348842	9.0	two-sided	0.210344	1.000000	bonf	0.631	0.610908
23	12	group * stim_type	1	v2	v3	True	True	-1.859568	9.0	two-sided	0.095879	1.000000	bonf	1.088	-0.251994
24	13	group * stim_type	2	f1	f2	True	True	0.915102	9.0	two-sided	0.384001	1.000000	bonf	0.436	0.133694
25	14	group * stim_type	2	f1	v2	True	True	-3.629391	9.0	two-sided	0.005491	0.065890	bonf	10.053	-0.293100
26	15	group * stim_type	2	f1	v3	True	True	-0.274779	9.0	two-sided	0.789690	1.000000	bonf	0.319	-0.040896
27	16	group * stim_type	2	f2	v2	True	True	-3.219512	9.0	two-sided	0.010498	0.125971	bonf	5.963	-0.448430
28	17	group * stim_type	2	f2	v3	True	True	-2.163063	9.0	two-sided	0.058774	0.705284	bonf	1.561	-0.188142

29	18	group * stim_type	2	v2	v3	True	True	2.089578	9.0	two-sided	0.066229	0.794744	bonf	1.428	0.276139

Stim_type = event type; Block = block of the sequence; Group = experimental groups; f1 = event F1; f2 = event F2; v2 = event V2; v3 = event V3; *p*-corr = *p* value.

Table S4: Significant *follow-up pairwise comparison* tests for data in the left side of Figure 5

	Contrast	fix	A	B	Paired	Parametric	T	dof	alternative	p-unc	p-corr	p-adjust	BF10	hedges
0	stim_type	-	f1	f2	True	True	-3.441234	9.0	two-sided	0.007376	0.044257	bonf	7.917	-1.148951
1	stim_type	-	f1	v2	True	True	-1.298951	9.0	two-sided	0.226251	1.000000	bonf	0.601	-0.407638
2	stim_type	-	f1	v3	True	True	-2.187394	9.0	two-sided	0.056490	0.338938	bonf	1.608	-0.617024
3	stim_type	-	f2	v2	True	True	1.427451	9.0	two-sided	0.187212	1.000000	bonf	0.681	0.610475
4	stim_type	-	f2	v3	True	True	1.051951	9.0	two-sided	0.320245	1.000000	bonf	0.484	0.435229

5	stim_type	-	v2	v3	True	True	-1.630891	9.0	two-sided	0.137350	0.824102	bonf	0.842	-0.173912
6	prev	-	v2	v3	True	True	-1.004880	9.0	two-sided	0.341206	NaN	NaN	0.467	-0.218038
7	stim_type * prev	f1	v2	v3	True	True	2.966780	9.0	two-sided	0.015785	0.063139	bonf	4.31	1.016544
8	stim_type * prev	f2	v2	v3	True	True	-3.638019	9.0	two-sided	0.005418	0.021670	bonf	10.163	-0.824656
9	stim_type * prev	v2	v2	v3	True	True	-1.339042	9.0	two-sided	0.213392	0.853567	bonf	0.625	-0.341446
10	stim_type * prev	v3	v2	v3	True	True	-0.353571	9.0	two-sided	0.731801	1.000000	bonf	0.326	-0.107397
11	prev * stim_type	v2	f1	f2	True	True	-0.466599	9.0	two-sided	0.651875	1.000000	bonf	0.339	-0.149808
12	prev * stim_type	v2	f1	v2	True	True	1.071455	9.0	two-sided	0.311855	1.000000	bonf	0.492	0.230145
13	prev * stim_type	v2	f1	v3	True	True	-0.274608	9.0	two-sided	0.789817	1.000000	bonf	0.319	-0.063966

14	prev * stim_type	v2	f2	v2	True	True	0.860810	9.0	two-sided	0.411687	1.000000	bonf	0.42	0.344645
15	prev * stim_type	v2	f2	v3	True	True	0.128866	9.0	two-sided	0.900298	1.000000	bonf	0.311	0.045984
16	prev * stim_type	v2	v2	v3	True	True	-1.132026	9.0	two-sided	0.286882	1.000000	bonf	0.518	-0.233029
17	prev * stim_type	v3	f1	f2	True	True	-4.506411	9.0	two-sided	0.001475	0.017704	bonf	29.572	-1.811698
18	prev * stim_type	v3	f1	v2	True	True	-2.216332	9.0	two-sided	0.053886	0.646628	bonf	1.666	-1.010067
19	prev * stim_type	v3	f1	v3	True	True	-3.090693	9.0	two-sided	0.012915	0.154980	bonf	5.054	-1.283177
20	prev * stim_type	v3	f2	v2	True	True	1.696057	9.0	two-sided	0.124110	1.000000	bonf	0.905	0.755842
21	prev * stim_type	v3	f2	v3	True	True	1.714065	9.0	two-sided	0.120662	1.000000	bonf	0.923	0.793824
22	prev * stim_type	v3	v2	v3	True	True	-0.192276	9.0	two-sided	0.851794	1.000000	bonf	0.314	-0.050715

Stim_type = event type; Prev = previous event; f1 = event F1; f2 = event F2; v2 = event V2; v3 = event V3; *p*-corr = *p* value.

Table S.5: Significant *follow-up pairwise comparison* tests for data in the right side of Figure 5

	Contrast	fix	A	B	Paired	Parametric	T	dof	alternative	p-unc	p-corr	p-adjust	BF10	hedges
0	stim_type	-	f1	f2	True	True	2.533318	9.0	two-sided	0.032060	0.192362	bonf	2.476	0.241356
1	stim_type	-	f1	v2	True	True	- 1.343266	9.0	two-sided	0.212074	1.000000	bonf	0.627	-0.162058
2	stim_type	-	f1	v3	True	True	- 0.596043	9.0	two-sided	0.565833	1.000000	bonf	0.359	-0.062503
3	stim_type	-	f2	v2	True	True	- 3.483199	9.0	two-sided	0.006904	0.041422	bonf	8.352	-0.412083
4	stim_type	-	f2	v3	True	True	- 2.283293	9.0	two-sided	0.048302	0.289810	bonf	1.809	-0.312567
5	stim_type	-	v2	v3	True	True	0.545966	9.0	two-sided	0.598360	1.000000	bonf	0.351	0.102426

6	prev	-	v2	v3	True	True	1.235319	9.0	two-sided	0.247986	NaN	NaN	0.567	0.110393
7	stim_type * prev	f1	v2	v3	True	True	2.830323	9.0	two-sided	0.019712	0.078848	bonf	3.617	0.250341
8	stim_type * prev	f2	v2	v3	True	True	1.386670	9.0	two-sided	0.198924	0.795698	bonf	0.654	0.145647
9	stim_type * prev	v2	v2	v3	True	True	- 2.745312	9.0	two-sided	0.022650	0.090599	bonf	3.244	-0.383232
10	stim_type * prev	v3	v2	v3	True	True	3.101411	9.0	two-sided	0.012694	0.050774	bonf	5.124	0.440918
11	prev * stim_type	v2	f1	f2	True	True	2.348552	9.0	two-sided	0.043408	0.520895	bonf	1.962	0.295292
12	prev * stim_type	v2	f1	v2	True	True	1.216993	9.0	two-sided	0.254555	1.000000	bonf	0.558	0.159414
13	prev * stim_type	v2	f1	v3	True	True	- 1.299438	9.0	two-sided	0.226091	1.000000	bonf	0.602	-0.141890
14	prev * stim_type	v2	f2	v2	True	True	- 1.636622	9.0	two-sided	0.136137	1.000000	bonf	0.848	-0.133786

15	prev * stim_type	v2	f2	v3	True	True	-2.671707	9.0	two-sided	0.025551	0.306611	bonf	2.952	-0.446230
16	prev * stim_type	v2	v2	v3	True	True	-1.954882	9.0	two-sided	0.082319	0.987824	bonf	1.216	-0.304885
17	prev * stim_type	v3	f1	f2	True	True	1.759258	9.0	two-sided	0.112394	1.000000	bonf	0.971	0.177043
18	prev * stim_type	v3	f1	v2	True	True	-3.572144	9.0	two-sided	0.006004	0.072049	bonf	9.35	-0.476633
19	prev * stim_type	v3	f1	v3	True	True	0.259522	9.0	two-sided	0.801072	1.000000	bonf	0.318	0.036503
20	prev * stim_type	v3	f2	v2	True	True	-3.688983	9.0	two-sided	0.005005	0.060063	bonf	10.838	-0.649401
21	prev * stim_type	v3	f2	v3	True	True	-1.247271	9.0	two-sided	0.243777	1.000000	bonf	0.573	-0.145651
22	prev * stim_type	v3	v2	v3	True	True	2.136542	9.0	two-sided	0.061365	0.736384	bonf	1.511	0.522497

Stim_type = event type; Prev = previous event; f1 = event F1; f2 = event F2; v2 = event V2; v3 = event V3; *p*-corr = *p* value.

Material S1: Statistical Analysis Details

Statistical Power Analysis

Statistical power was estimated using pilot data from individual participants, based on sample means and variances of reaction time distributions and assuming Gaussian-shaped distributions. Worst- and best-case scenarios were considered by varying effect size, variance, and sample size. Under these assumptions, estimated statistical power ($1-\beta$) ranged from 0.07 to 0.85 for the Execution group and from 0.13 to 0.99 for the Motor Imagery group, indicating that, despite the limited sample size, the design had the potential to detect meaningful effects while remaining exploratory in nature. Power analyses were conducted using custom Python scripts to ensure transparency and reproducibility (the code for the statistical power analysis is in: https://github.com/PauloCabral-hub/third_party_work/blob/main/statistical_power_analysis.ipynb).

Figure 3

In the analysis described for the figure, before the 2-way repeated measures ANOVA, the distributions of each event and block were tested for normality and sphericity adopting a significance level of 0.01. The Shapiro-Wilk test indicated a violation of normality in block 1 [33]. Given that, the values of the distributions were rank-transformed and tested for normality again. After transformation, no violation was detected. The p-values of the 2-way repeated measures ANOVA were corrected using Greenhouse–Geisser correction in case of violations of sphericity [34].

Figure 4

In the analysis described for the figure, before applying the 2–way mixed ANOVA, the distributions were tested for normality, homogeneity of variance, and sphericity. Given the violation in normality indicated by the Shapiro-Wilk test, the values were rank transformed and tested again for the three assumptions. The Levene test was used to test the homogeneity of variance [35].

Figure 5

In the analysis described for the figure, there were no indications of violation of normality by the Shapiro-Wilk test. Violations of sphericity were treated with Greenhouse-Geisser correction.
